# Rapid on-site evaluation of the development of resistance to quinone outside inhibitors in *Botrytis cinerea*

**DOI:** 10.1038/s41598-017-13317-z

**Published:** 2017-10-24

**Authors:** X. R. Hu, D. J. Dai, H. D. Wang, C. Q. Zhang

**Affiliations:** 1 Department of Plant Pathology, Zhejiang Agriculture and Forest University, Lin’an, 311300 China; 2 Institute for the Control of Agrochemicals of Zhejiang Province, Hangzhou, 310020 China

## Abstract

*Botrytis cinerea*, a typical “high-risk” pathogenic fungus that rapidly develops resistance to fungicides, affects more than 1,000 species of 586 plant genera native to most continents and causes great economic losses. Therefore, a rapid and sensitive assay of fungicide resistance development in *B. cinerea* populations is crucial for scientific management. In this study, we established a Loop-mediated isothermal amplification (LAMP) system for the monitoring and evaluation of the risk of development of *B. cinerea* resistance to QoI fungicides; the method uses two LAMP assays. The first assay detects G143A mutants of *B. cinerea*, which are highly resistance to QoI fungicides. BCbi143/144 introns in *B. cinerea* are then detected by the second assay. HNB acts as a visual LAMP reaction indicator. The optimum reaction conditions of the LAMP assays were 61 °C for 50 min, and the detection limit of the LAMP assays was 100 × 10^−4^ ng/μl. We directly pre-treated the field samples by using All-DNA-Fast-Out to extract DNA within ten minutes, then performed the LAMP assay to achieve one-step rapid detection. In conclusion, we established a rapid and sensitive LAMP assay system for resistance risk assessment and for monitoring QoI-resistance of *B. cinerea* in the field.

## Introduction

Gray mold is caused by *Botrytis cinerea* Pers:Fr., which is one of the most economically important pathogens of strawberry plants^1,2^. The infection may occur in the flower, maintain latency until fruit maturation, and develop abundantly into visible infection, causing fruit rot accompanied by profuse sporulation during harvest operations^3^. The management of gray mold disease is achieved by frequent application of fungicides. Quinone outside inhibitors (QoIs) were developed on the basis of the natural products of β-methoxyacrylate acid, including strobilurin A and oudemansin A^4^. QoIs commonly used in agricultural production include pyraclostrobin and azoxystrobin^5^. The activity of QoIs is derived from the ability to bind to the Qo site in cytochrome b in fungi, thereby inhibiting mitochondrial respiration^6^. Cytochrome b is part of the cytochrome bc1 complex located in the mitochondrial inner membrane. After an inhibitor is bound to cytochrome b, it prevents electron transfer between cytochrome b and c1. Because QoIs have a wide range of control efficiency for most important fungal diseases in agriculture, they have become crucial parts of plant disease management practices^5^. Unfortunately, strains highly resistant to QoIs have been reported for different target pathogens, such as *Podosphaera fusca* ^7^, *Venturia inaequalis* ^8^, *Mycosphaerella graminicola* ^9,10^, *Plasmopara viticola* ^11,12^, *Erysiphe necator* ^12^, *Colletotrichum graminicola* ^13^, *and B. cinerea* ^14^. In general, a point mutation at codon 143 of *cytb* gene results in a substitution of glycine by alanine (G^143^ → A) in plant-pathogenic fungi, thus causing resistance to QoIs^15,16^. In addition, isolates of *B. cinerea* can be divided into two types: those with or without the Bcbi-143/144 intron in *cytb* ^14^. QoI-resistant isolates have been found with the G143A mutation but without the Bcbi-143/144 intron. Isolates with Bcbi-143/144 introns are very low-risk for development of resistance to QoIs. This phenomenon has also been reported for other plant pathogens^17–19^.

The traditional method of detection or monitoring of fungicide resistance is the minimum inhibitory concentration (MIC) accompanied by long detection cycles, of a week or even longer^20,21^. In recent years, with the development of nucleic acid-related molecular detection, PCR-based detection techniques have been developed^22,23^. However, these techniques have inherent shortcomings, including the prolonged time and expensive equipment required, thus limiting these methods to laboratories and making them unsuitable for field testing. To achieve rapid detection, Loop-mediated isothermal amplification (LAMP) is now essential for pathogen detection, because it is specific, efficient and fast^24,25^. In this study, LAMP assays were established for the detection of *B. cinerea* resistance to QoI fungicide and the susceptible strain containing BCbi143/144 introns for risk assessment and monitoring of resistance to QoIs in populations of *B. cinerea*.

## Results

### Visual detection of two LAMP reactions

**Figure 1:**
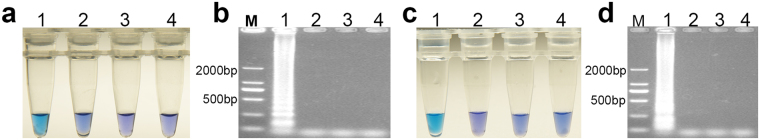
Specificity detection of LAMP assay. (**a**) Specificity detection of G143A-LAMP on the basis of HNB color change, label 1: LAB12-06, 2: TMB15-06, 3: ANB13-07, 4: ddH_2_O. (**b**) Specificity detection of G143A-LAMP based on gel electrophoresis, label 1: LAB12-06, 2: TMB15-06, 3: ANB13-07, 4: ddH_2_O. (**c**) Specificity detection of BCbi143/144-LAMP on the basis of HNB color change, label 1: ANB13-07, 2: LAB12-06, 3: TMB15-06, 4: ddH_2_O. (**d**) Specificity detection of G143A-LAMP on the basis of gel electrophoresis label 1: ANB13-07, 2: LAB12-06, 3: TMB15-06, 4: ddH_2_O.

For DNA samples extracted from the G143A genotype of *B. cinerea* strains, the specific G143A-LAMP mismatched primers S7 (Table 1) were screened, and the G143A mutants were specifically detected; the positive reaction was reflected in the color change from violet to sky blue according to HNB (Fig. 1a). The products of the G143A mutants showed a ladder-like pattern in the gel electrophoresis (Fig. 1b). Furthermore, positive samples with the BCbi143/144 intron were amplified by another specific BCbi143/144-LAMP primer set S9 (Table 1) for the BCbi143/144-LAMP assay, also based on HNB color changes to determine the extent of reaction (Fig. 1c) and by gel electrophoresis (Fig. 1d). As expected, all positive samples showed significant color changes from violet to sky blue according to HNB, but the negative controls did not (Fig. 1).

**Table 1:** Primers used in this study.

Primer name	Primer set name	Type	Sequence (5′–3′)
G143A-F3		Forward outer	TGTATGTTCTGCCCTACG
G143A-B3		Reverse outer	CCTTTAGATGTTCTGG
G143A-FIP1	S1	Forward inner	GTCCAATTCATGGTACAGCACGGCAAATGTCACTGTGAGCT
G143A-FIP2	S2	Forward inner	GTCCAATTCATGGTACAGCACGGCAAATGTCACTGTGATC
G143A-FIP3	S3	Forward inner	GTCCAATTCATGGTACAGCACGGCAAATGTCACTGTGACC
G143A-FIP4	S4	Forward inner	GTCCAATTCATGGTACAGCACGGCAAATGTCACTGTGAAC
G143A-FIP5	S5	Forward inner	GTCCAATTCATGGTACAGCACGGCAAATGTCACTGTGATCA
G143A-FIP6	S6	Forward inner	GTCCAATTCATGGTACAGCACGGCAAATGTCACTGTGATCC
G143A-FIP7	S7	Forward inner	GTCCAATTCATGGTACAGCACGGCAAATGTCACTGTGTTC
G143A-FIP8	S8	Forward inner	GTCCAATTCATGGTACAGCACGGCAAATGTCACTGTGGTC
G143A-BIP		Reverse inner	GATATTGTTGAGTCAAACAACCCATCTCCATCCACCATACC
BCbi143/144-F3	S9	Forward outer	CCTAATCAAATGGCTAAACGTATT
BCbi143/144-B3		Forward outer	CGTACAGTAACCATGGGATA
BCbi143/144-FIP		Forward inner	TGAGAATCACCTAAGAGTGAACCATGCTTTTAAACGAATAGGACCG
BCbi143/144-BIP		Reverse inner	CCGATTACATGGAAACGGAACTCAAAAGTCATGCAGTCACAAT

### Optimal running-conditions for two LAMP assays

**Figure 2:**
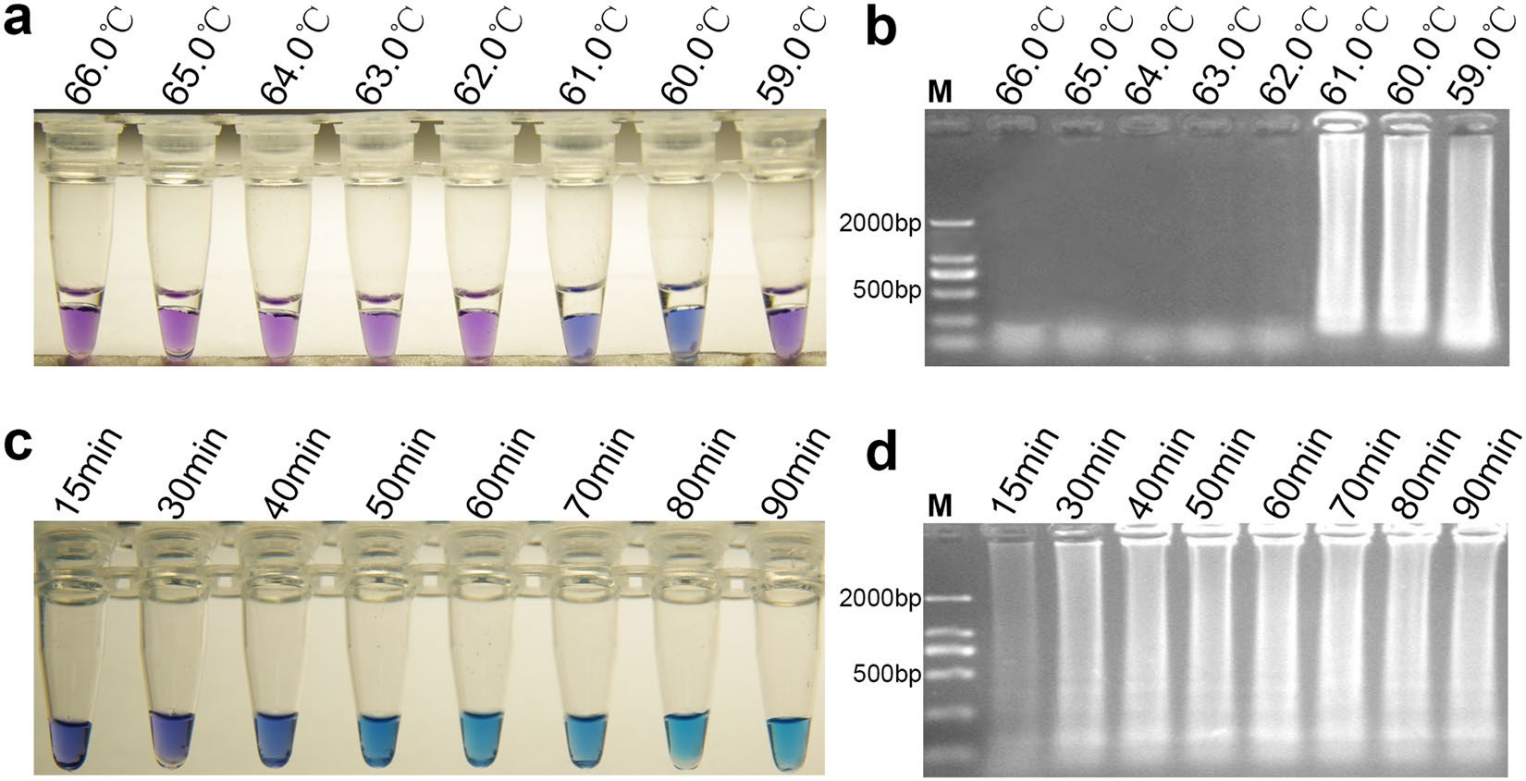
Optimization of reaction conditions for G143A – LAMP. Reaction temperature gradient of the G143A-LAMP was set to 59.0 °C, 60.0 °C, 61.0 °C, 62.0 °C, 63.0 °C, 64.0 °C, 65.0 °C, 66.0 °C. (**a**) Optimization of temperature on the basis of HNB color change. (**b**) Optimization of temperature gradient on the basis of gel electrophoresis detection. Reaction time of G143A-LAMP was set to 15 min, 30 min, 40 min, 50 min, 60 min, 70 min, 80 min and 90 min. (**c**) Optimization of reaction time on the basis of HNB color change. (**d**) Optimization of reaction time on the basis of gel electrophoresis detection.

**Figure 3:**
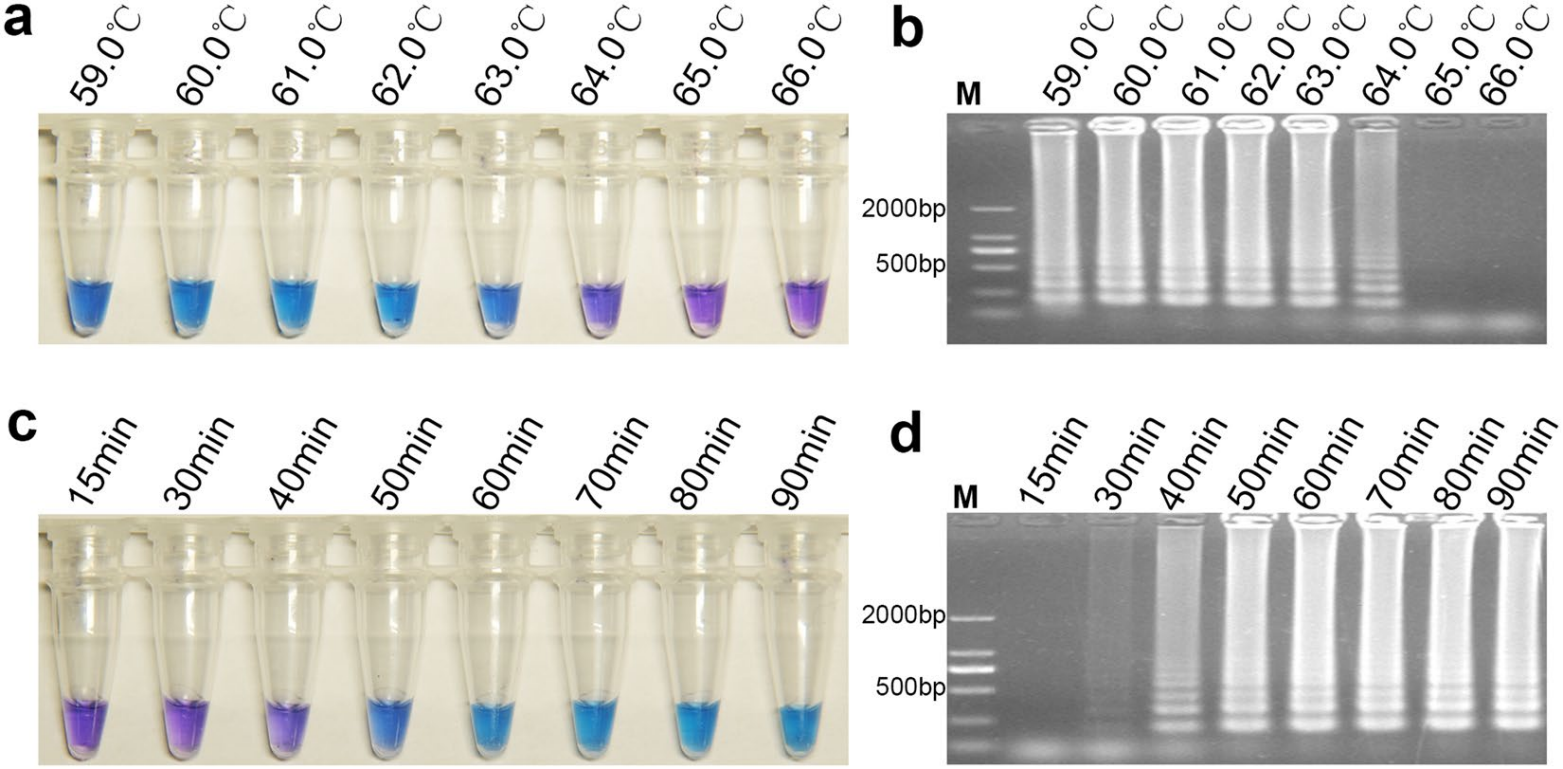
Optimization of reaction conditions for BCbi143/144-LAMP. Reaction temperature gradient of BCbi143/144-LAMP were set to 66.0 °C, 65.0 °C, 64.0 °C, 63.0 °C, 62.0 °C, 61.0 °C, 60.0 °C, 59.0 °C. (**a**) Optimization of temperature gradient on the basis of HNB color change. (**b**) Optimization of temperature on the basis of gel electrophoresis detection. Reaction times of BCbi143/144-LAMP were set to 15 min, 30 min, 40 min, 50 min, 60 min, 70 min, 80 min, 90 min. (**c**) Optimization of reaction time on the basis of HNB color change. (**d**) Optimization of reaction time on the basis of gel electrophoresis detection.

The optimal running conditions for LAMP reactions using a specific primer set were determined by HNB visualization and gel electrophoresis analysis of amplification products. The results clearly indicated that the G143A-LAMP reaction could not be performed and that the color of the reaction tube did not change when the temperature was higher than 61 °C (Fig. 2a). However, the reaction mixture showed significant color changes, and clear LAMP bands were displayed in the gel electrophoresis at 60 °C and 61 °C (Fig. 2a,b). Therefore, to optimize the reaction time, G143A-LAMP was performed at 61 °C. The results showed that suitable detection could be achieved at 61 °C for 50 min (Fig. 2c,d). Another LAMP assay to detect the BCbi143/144 intron in *B. cinerea* successfully proceeded at 59 °C to 63 °C, and was judged by color change or gel electrophoresis (Fig. 3a,b). Thus, 61 °C was selected as the reaction temperature to support both LAMP assays. The optimum reaction condition of this LAMP was 61 °C for 50 min (Fig. 3c,d).

### Sensitivity of LAMP

**Figure 4:**
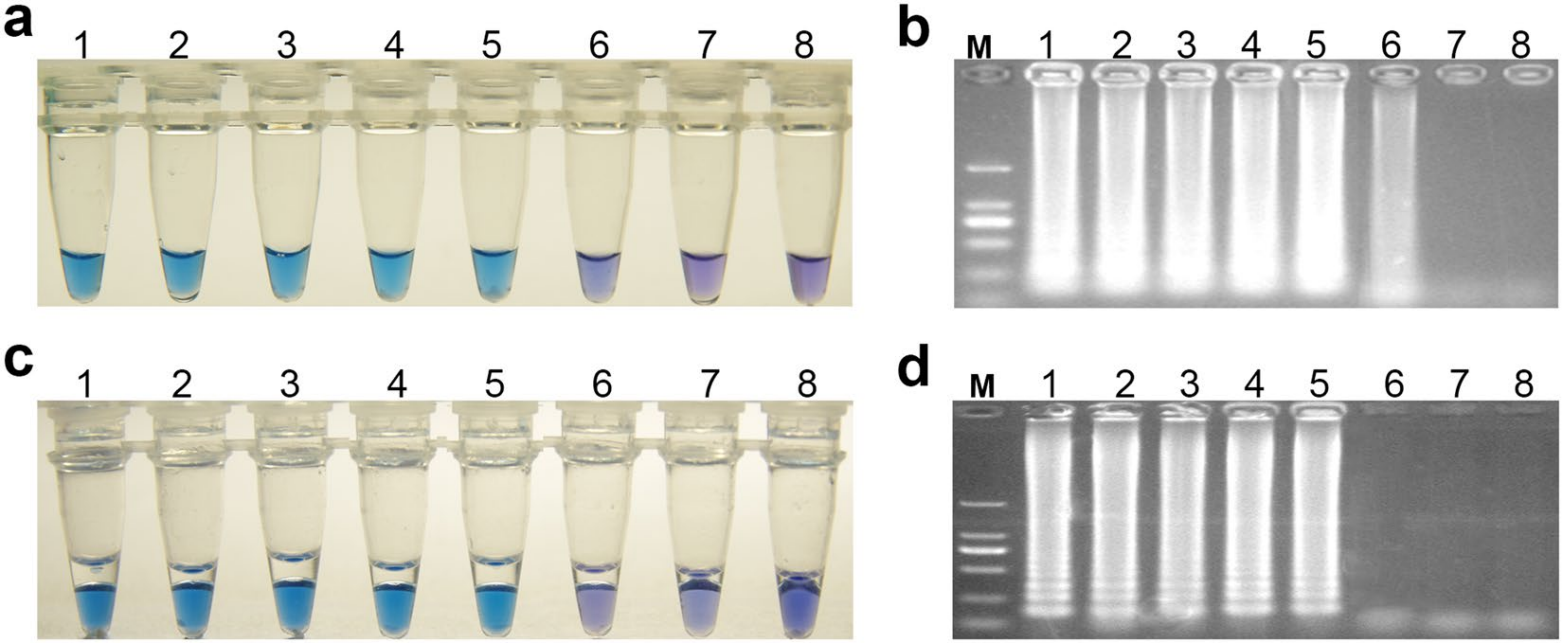
Sensitivity test of G143A-LAMP and BCbi143/144-LAMP. Reaction tubes 1–7 were 10-fold dilutions of the cleavage products, with DNA concentrations of 100, 100 × 10^−1^, 100 × 10^−2^, 100 × 10^−3^, 100 × 10^−4^, 100 × 10^−5^, 100 × 10^−6^ ng/μl, and the tube DW was ddH_2_O blank control. (**a**) Sensitivity detection on the basis of HNB color change. (**b**) Sensitivity detection on the basis of gel electrophoresis detection.

For the sensitivity test, 10-fold diluted DNA samples were used as templates for LAMP sensitivity testing on the basis of the visible color change of HNB in the tube (Fig. 4a,c) and the results from gel electrophoresis (Fig. 4b,d). The detection limit of the G143A-LAMP assay and BCbi143/144-LAMP assay was 100 ×  10^−4^ ng/μl.

### On-site detection of strawberry samples within 1 h

**Figure 5:**
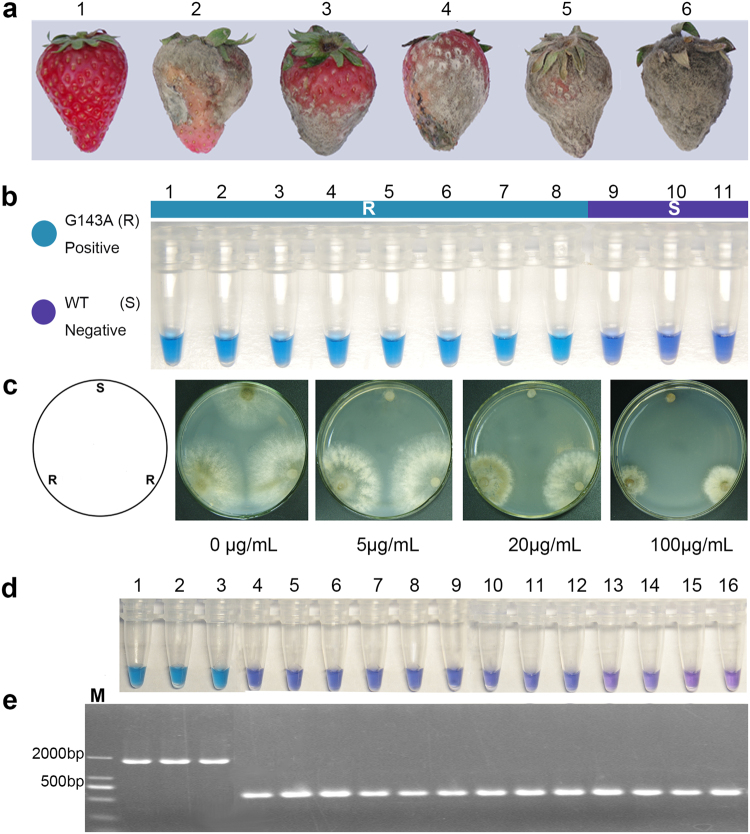
Detection of field strawberry samples by LAMP assays in fields and MIC testing in laboratory. (**a**) Strawberry samples from field, label 1 healthy samples, label 2–6 strawberry friuts of gray mold in field. (**b**) G143A-LAMP detection based on the color change of HNB, label 1–8 G143A mutant phenotypes of *B. cinere*a, labels 9–11: no mutant strains of *B. cinerea*. (**c**) Detection of field samples by MIC method, S representes QoI-sensitive strain, R representes strain of QoI-resistance. (**d**) Bcbi 143/144-LAMP reaction causing change in color, lables 1–3: *B.cinerea* with Bcbi –143/144 intron, lables 4–16: *B.cinerea* without Bcbi –143/144 intron. (**e**) Detection of the intron of the strain using PCR amplification.

All field samples were treated with All-DNA-Fast-Out at 80 °C for 5–10 minutes to extract genomic DNA. The supernatant obtained from the lysate was directly added to the LAMP mixture as DNA template. The LAMP results of the G143A assay showed 46 positive reactions from 78 samples. According to the MIC test, 46 isolates with high resistance to QoI fungicides were detected with an MIC > 100 μg ml^−1^ (Fig. 5), and the frequency of resistant strains was 59% (a total of 78), as shown in Table 2. PCR sequencing analysis indicated that all isolates highly resistant to azoxystrobin had the G143A mutation in the *cytb* gene (Table 2). The G143A-LAMP assay specifically detected isolates with the G143A genotype with 100% accuracy (Fig. 5, Table 2). For the BCbi143/144-LAMP assay, three of the 78 field samples were positive. The PCR amplification results were consistent with those of the BCbi143/144-LAMP assay. In the gel electrophoresis (Fig. 5), there were two types of *cytb* in *B. cinerea*: type I was followed by an intron (1205 bp) at codon 143, and the products amplified by primers BC-cytb-F and BC-cytb-R were 1700 bp (3 samples); for type II, the 143^rd^ codon was not followed by an intron, and the products were approximately 550 bp with primers BC-cytb-F and BC-cytb-R.

**Table 2:** Field sample testing with LAMP, MIC and PCR.

Origin	Number of samples	LAMP Positive		MIC Positive^x^	Number of t mutation genotypes	
		G143A-LAMP	BCbi143/144-LAMP		G143A	BCbi143/144 intron
Jiashan, zhejiang	14	3	0	3	3	0
Jiande, Zhejiang	11	4	0	4	4	0
Xiasha, Zhejiang	10	7	0	7	7	0
Zhuji, Zhejiang	10	9	1	9	9	1
Linan, Zhejiang	21	12	2	12	12	2
Tongxiang, Zhejiang	12	11	0	11	11	0
Total	78	46	3	46	46	3

^X^MIC Positive indicates a MIC (minimum inhibitory concentration) >100 μg ml^−1^.

## Discussion

LAMP is an innovative technique for gene amplification and a simple diagnostic tool for the early detection and identification of diseases^26,27^. LAMP provides a simpler method of diagnosing pathogenic fungi^28,29^ and can has the specificity sufficient to detect single-base differences in DNA fragments^30–32^. In the current research, mismatched primers were designed and screened to detect QoI-resistance in *B. cinerea*. For this purpose, mismatched bases were introduced at the 3′ end of FIP to separate G143A mutant genotype strains from sensitive strains. Eight sets of primers were screened, and primers S7 with a change in the second and third nucleotide at the 3′end of FIP (from A and G to T and T) specifically detected G143A *B. cinerea*. Additionally, another primer set for intron detection were designed according to the 1205 bp of the BCbi143/144 intron sequence and was used to monitor and evaluate the low-resistance risk of a *B. cinerea* population. In this study, a visual color change to sky blue from violet indicated HNB in positive samples through the LAMP system, whereas negative samples remained violet. This sealed reaction decreased the risk of false positives observed when the DNA-intercalating dye SYBR Green is added after amplification^33,34^.

The detection method of *B. cinerea* resistance to QoIs was designed to meet some of the main requirements for on-site rapid testing. To make LAMP suitable for field diagnostics^28,30^, pretreatment of samples must be as simple as possible. In the current one-step LAMP, samples were pre-treated with All-DNA-Fast-Out to extract DNA, and the lysates, as DNA templates, were added to prepared LAMP reaction mixtures and incubated in a heated block. The procedure is sufficiently simple to potentially allow this on-site assay to be performed without precise equipment and experienced staff. Conventional nucleic acid-based methods have many equipment requirements, such as liquid nitrogen, centrifuges and expensive thermocyclers with a series of different temperatures to achieve amplification in techniques such as AS-PCR, PCR-RFLP, real-time PCR, and RAPD-PCR^22,23^. Compared with these existing PCR-based detection methods, this assay is relatively simple and generates easily interpreted results in just over 1 hour, including the time required for DNA extraction. A variety of field samples were assessed by using All-DNA-Fast-Out in a single step (approximately 5–10 minutes). The supernatant of the lysate can be directly used for LAMP amplification without centrifugation, extraction or other operational steps for traditional DNA extraction^31,32^, thereby minimizing sample pretreatment time and decreasing contamination between samples to achieve rapid detection.

The *B. cinerea* population constitutes three sub-populations: resistant to QoIs (common of G143A), sensitive to QoIs with Bcbi-143/144 intron and sensitive to QoIs without Bcbi-143/144 intron^14,15^. The QoI-sensitive isolates without the Bcbi-143/144 intron have a high risk of developing resistance to QoI through the G143A single mutation, which is the main resistance mechanism of resistance to QoIs in *B. cinerea*. In contrast, the QoI-sensitive isolates with the Bcbi-143/144 intron generally have very low risk of developing resistance to QoIs^18,19^. In this study, to evaluate the use of LAMP as a field diagnostic tool for the application of QoIs against gray mold disease, we established a LAMP system for monitoring and evaluation of the risk of *Botrytis cinerea* resistance to QoIs. G143A-LAMP detected isolates resistant to QoIs, and BCbi143/144-LAMP indicated the percentage of the sub-population with a very low risk of resistance development. In general, gray mold results in 20 to 30% yield loss, up to 50% when the environment is favorable for *B. cinerea* epidemics. The management of gray mold is somewhat reliant on frequent applications of fungicides^35–37^. QoI should not be used anymore for the management of *B. cinera* with serious resistance as the tested populations in this study. According the results of these two assays, however, QoIs can be applied in the scientific and sustainable management of *B. cinerea* in context with low or without resistance has occurred.

## Materials and Methods

### Isolates for developing LAMP assays

Three isolates of *B. cinerea* were adopted to develop the LAMP assays. ANB13-O7 is the type with the Bcbi-143/144 intron inserted between the 143^rd^ and 144^th^ codon in the cytb gene and is sensitive to azoxystrobin. LAB12-06 is highly resistant to azoxystrobin and has a point mutation at codon 143 in the cytb gene (G143A). TMB15-06 contains the *cytb* gene without the Bcbi-143/144 intron and is sensitive to azoxystrobin. Mycelia (approximately 2 mg) of *B. cinerea* were collected from each purely cultured strain on PDA, placed in a 0.2 ml tube containing 50 μl of DNA-EZ Reagents V All-DNA-Fast-Out (Sangon, Shanghai) and heated at 80 °C for 5 min in a water bath. The lysed supernatant was used as a DNA template to develop the LAMP assay. The extracted DNA was quantified by spectrophotometry and diluted with distilled water.

### LAMP primers design and screening

**Figure 6:**
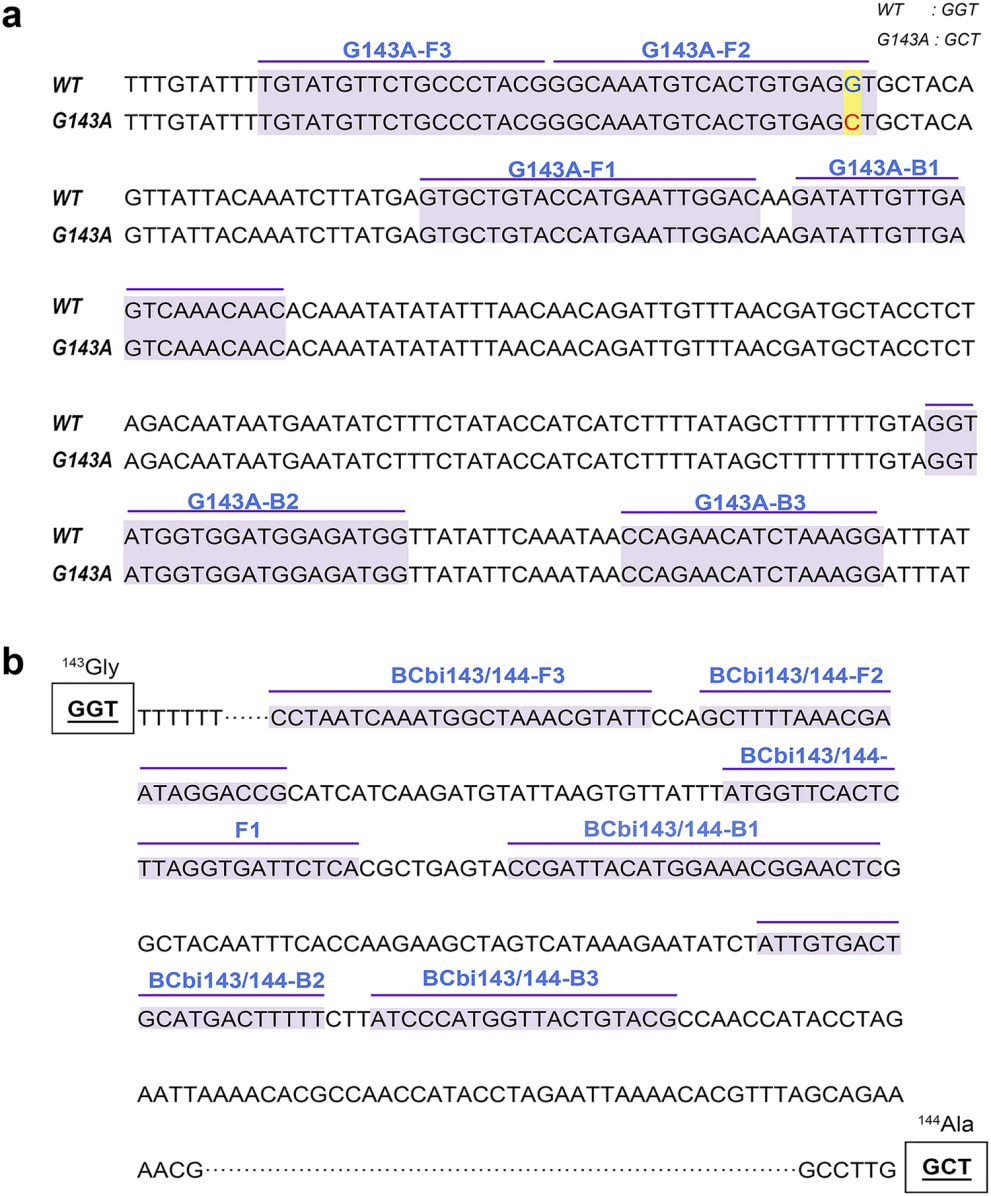
Location of LAMP primers in Cytochrome b gene of *B. cinerea*. (**a**) LAMP primers used to detect G143A mutant genotype. (**b**) LAMP primers used to detect BCbi143/144 intron of cytb gene. Bold lines and shadows indicate primer location.

In the current study, two LAMP tests were developed. One LAMP assay, referred to as the G143A-LAMP assay, detected G143A mutants of *B. cinerea*, which is highly resistant to QoI fungicides. The G143A-LAMP primers were designed and mismatched on the basis of the point mutation at codon 143 (G143A) in the cytb gene by using Primer explorer V5 software (http://primerexplorer.jp/e/). Mismatched bases were introduced at the 3′ end of the FIP to separate G143A genotype strains from sensitive strains. Several sets of mismatched LAMP primers were screened from specificity and sensitivity to select a set of optimal primers. In addition, to detect the presence of the BCbi143/144 intron in *B. cinerea*, another LAMP primer set was designed according to the intron 1205 bp DNA sequence and a unique 200–300 bp sequence from this intron, referred to as BCbi143/144-LAMP assay. The LAMP primer sequences used are shown in Fig. 6 and Table 1.

### LAMP reaction mixtures

Each LAMP assay was performed in a 25-μl reaction mixture, including a final concentration of 8 μl Bst DNA polymerase (New England Biolabs, Beijing), 2.5 μl 10 × ThermoPol buffer, 1 mM dNTPs, 5 mM Mg^2+^, 1.6 μM FIP and BIP, 0.2 μM F3 and B3, 0.6 M betaine, 150 μM hydroxynaphthol blue (HNB, metal ion indicator), and 1 μl DNA sample (a concentration of 100 ng/μl that extracted by All-DNA-Fast-Out), and the volume was adjusted to 25 μl with nucleic acid-free water. In the G143A-LAMP assay, DNA samples extracted from the G143A mutant genotype (LAB12-06) were used as positive samples, and TMB15-06 with no mutation at codon 143 was selected as a negative control, and nucleic acid-free water was used as a blank control. For the BCbi143/144-LAMP assay, isolate ANB13-07 was used as a positive control, and negative samples of TMB15-06 were used. Both LAMP reactions were incubated at 61 °C for 60 min and 80 °C for 5 min. Each treatment was repeated at least three times, as below. The reaction results were examined via visual color changes of HNB (from violet to sky blue) after the reaction and/or further confirmed via 1% agarose gel electrophoresis.

### Determination of running-conditions

After the LAMP reactions were complete, the HNB color change in the reaction mixture was observed by naked eye under sunlight. For positive results, sky blue with HNB was observed, and negative or blank treatments remained violet. If confirmation was required, the reaction products (5 μl) were further assayed by 1% agarose gel electrophoresis, and the positive results showed typical LAMP ladder-like bands; negative reactions lacked these bands. To determine the optimal running conditions for the reactions, we set a series of constant temperatures, e.g., 59.0, 60.0, 61.0, 62.0, 63.0, 64.0, 65.0, 66.0 °C to detect the optimal reaction temperature. The LAMP assay was performed for 15, 30, 40, 50, 60, 70, 80, and 90 min at the optimal reaction temperature to determine the shortest optimal time.

### Detection limit of LAMP assay

From the PDA surface of a pure culture of *B. cinerea*, hyphae were picked to extract DNA with ALL-DNA-Fast-Out. DNA samples were 10-fold diluted in water and used as templates for LAMP sensitivity testing; the final concentration of DNA samples was 100, 100 × 10^−1^, 100 × 10^−2^, 100 × 10^−3^, 100 × 10^−4^, 100 × 10^−5^, and 100 × 10^−6^ ng/μl. The detection limit represented the lowest DNA concentration at which positive results were observed. Samples were observed for HNB color change and further analyzed by 1% agarose gel electrophoresis.

### Application to on-site rapid detection

To assess this LAMP for on-site detection, a total of 78 diseased strawberry fruits from greenhouses in six different geographical regions in Zhejiang Province, China during 2017 were tested. For each fruit, approximately 2 mg mold was collected from the fruit surface and added to 0.2 ml PCR tubes containing 50 μl of All-DNA-Fast-Out (Sangon, Shanghai). After incubation at 80 °C for 5–10 min in a heated block, the supernatant obtained was directly used in the LAMP assay as described above. Furthermore, each tested strawberry fruit was taken to the laboratory to verify the results of the on-site LAMP detection. *B. cinerea* was isolated and purified from a single colony isolated from the 78 strawberry samples. The sensitivity to azoxystrobin was evaluated using the traditional method of minimum inhibitory concentration (MIC). Briefly, mycelial plugs (5 mm) of each isolate were placed on potato dextrose agar (PDA) plates with a series of 0, 5, 20, and 100 μg ml^−1^ azoxystrobin (Syngenta, China). Each fungicide concentration was treated three times. All plates were incubated at 23 °C for 3 d. If the isolate had an MIC > 100 μg ml^−1^, it was designated as highly resistant to QoIs^21^. The DNA fragment including the 143^rd^ codon of the *B. cinerea* cytb gene was amplified by conventional PCR using the designed primers BC-cytb-F (5′-TAAAGTGGTATAACCCGACGG-3′) and BC-cytb-R (5′-CCATCTCCATCCACCATACCT-3′). The reactions contained the following reagents: 25 μl 2 × PCR Master, 0.4 μM primers of BC-cytb-F and BC-cytb-R, 1 μl DNA template, and ddH_2_O to adjust the volume to 50 μl. The thermal cycling of the conventional PCR program was 95 °C for 5 min; 30 reaction cycles of 95 °C for 30 s, 55 °C for 30 s, and 72 °C for 90 s with an extension at 72 °C for 5 min. The PCR products were separated by 1% agarose gel electrophoresis, and genomic DNA was purified using a UNIQ-10 Column DNA Purification Kit (Sangon, Shanghai). The purified product was directly inserted into the T-Vector PMD-19 (TAKALA, Dalian), according to the manufacturer’s instructions. All purified PCR products were sequenced by Sangon, Shanghai. The resulting sequences were aligned using Clustal W software. (http://www.bi.ac.uk).
